# Gene expression patterns of sulfur starvation in *Synechocystis *sp. PCC 6803

**DOI:** 10.1186/1471-2164-9-344

**Published:** 2008-07-21

**Authors:** Zhigang Zhang, Ninad D Pendse, Katherine N Phillips, James B Cotner, Arkady Khodursky

**Affiliations:** 1 BioTechnology Institute, 1479 Gortner Avenue, University of Minnesota, St. Paul, MN 55108, USA; 2 Department of Chemical Engineering and Materials Science, 421 Washington Avenue SE, University of Minnesota, Minneapolis, MN 55455, USA; 3 Department of Ecology, Evolution and Behavior, 1987 Upper Buford Circle, University of Minnesota, St. Paul, MN 55108, USA; 4 Department of Biochemistry, Molecular Biology and Biophysics, 1479 Gortner Avenue, University of Minnesota, St. Paul, MN 55108, USA; 5 InVivoScribe Technology, 6330 Nancy Ridge Drive, Suite 106, San Diego, CA 92121, USA

## Abstract

**Background:**

The unicellular cyanobacterium *Synechocystis *sp. PCC 6803 is a model microbe for studying biochemistry, genetics and molecular biology of photobiological processes. Importance of this bacterium in basic and applied research calls for a systematic, genome-wide description of its transcriptional regulatory capacity. Characteristic transcriptional responses to changes in the growth environment are expected to provide a scaffold for describing the *Synechocystis *transcriptional regulatory network as well as efficient means for functional annotation of genes in the genome.

**Results:**

We designed, validated and used *Synechocystis *genome-wide oligonucleotide (70-mer) microarray (representing 96.7% of all chromosomal ORFs annotated at the time of the beginning of this project) to study transcriptional activity of the cyanobacterial genome in response to sulfur (S) starvation. The microarray data were verified by quantitative RT-PCR. We made five main observations: 1) Transcriptional changes upon sulfate starvation were relatively moderate, but significant and consistent with growth kinetics; 2) S acquisition genes encoding for a high-affinity sulfate transporter were significantly induced, while decreased transcription of genes for phycobilisome, photosystems I and II, cytochrome b_6/f_, and ATP synthase indicated reduced light-harvesting and photosynthetic activity; 3) S starvation elicited transcriptional responses associated with general growth arrest and stress; 4) A large number of genes regulated by S availability encode hypothetical proteins or proteins of unknown function; 5) Hydrogenase structural and maturation accessory genes were not identified as differentially expressed, even though increased hydrogen evolution was observed.

**Conclusion:**

The expression profiles recorded by using this oligonucleotide-based microarray platform revealed that during transition from the condition of plentiful S to S starvation, *Synechocystis *undergoes coordinated transcriptional changes, including changes in gene expression whose products are involved in sensing nutrient limitations and tuning bacterial metabolism. The transcriptional profile of the nutrient starvation was dominated by a decrease in abundances of many transcripts. However, these changes were unlikely due to the across-the-board, non-specific shut down of transcription in a condition of growth arrest. Down-regulation of transcripts encoding proteins whose function depends on a cellular S status indicated that the observed repression has a specific regulatory component. The repression of certain S-related genes was paralleled by activation of genes involved in internal and external S scavenging.

## Background

Genome-wide surveys of transcript abundances in different conditions have become a major strategy toward delineating transcriptional regulation in many organisms. While there is an infinite number of conditions and their combinations, certain conditions can be viewed as essential and our understanding of transcriptional regulation in such conditions is critical for associating macroscopic phenotypes with regulatory pathways. Such essential conditions include, but are not limited to, variations in nutrient composition of the growth environment. Indeed, bacterial growth depends on the availability of macronutrients that can serve as sources of carbon (C), nitrogen (N) phosphorus (Pi), and sulfur (S). In the absence of any one of these macronutrients in the environment, cells cease to grow and divide. While it is not surprising that biomass increase, which is prerequisite for normal growth, cannot happen without sufficient continued supply of nutrients, the regulatory consequences of nutritional challenges are not trivial. Understanding such effects on the molecular level, which would involve characterizing both condition-specific and condition-independent regulatory patterns, is especially important when dealing with reversible growth arrest or with secondary metabolic processes triggered by nutrient limitation or starvation. One such secondary process is hydrogen production by cyanobacteria.

The fresh-water, unicellular non-nitrogen-fixing cyanobacterial strain *Synechocystis *sp. PCC 6803 (hereinafter *Synechocystis*) is emerging as a model for photobiological production of hydrogen, a clean and sustainable energy carrier [1]. Under certain conditions, this oxygenic photosynthetic organism can use reductant from water-splitting photosynthetic processes to generate molecular hydrogen [2-5]. The derived protons and electrons can be redirected to drive H_2 _production via the bidirectional hydrogenase, typically characterized by its sensitivity to oxygen [6,7]. Cyanobacterial hydrogen production is known to increase substantially under conditions of nutrient limitation, including N and S limitation [5,6,8,9]. It has been suggested that limitation of macronutrients could result in accumulation of carbon that can then be used for H2 production. However, the details of the bacterial H_2 _response to nutrient limitations and its regulation are yet to be determined. In principle, it would be impossible to optimize H_2 _production without systematic understanding of the interactions between hydrogen metabolism, respiration, photosynthesis and bacterial nutritional status. Additionally, an understanding of the details of bacterial metabolism under various macro- and micronutrient limitations will help elucidate the effect of such interactions on the ecology of bacteria under natural conditions.

Availability of the complete genome sequence of *Synechocystis *sp. PCC 6803 [10-12] offers an opportunity to develop technology to study and optimize metabolic and regulatory interactions in this bacterium. The genome is approximately 3.6 M bp long. It contains more than 3200 chromosomal protein coding genes and slightly over 50 genes encoding structural RNAs [13]. Expression profiling by DNA microarray technology [14,15] has enabled tremendous advances in our ability to understand genome-wide regulatory and metabolic processes in a diversity of organisms. This technology has provided insights into cyanobacterial transcriptional responses such as in acclimation to high light [16], salt stress [17], and nutrient limitations [18-22].

Here, we present a design and application of the genome-wide *Synechocystis *microarray. First, we developed and validated a new DNA microarray platform, based on long oligonucleotide probes (70-mer) spotted on a glass support. We chose to build an oligo-based array because it allowed us to circumvent the labor-intensive and error-prone steps of probe amplification and purification. Additionally, the quality of cDNA arrays may vary between PCR batches, which would affect researchers' ability to standardize data across the experimental sets. The specificity of and hybridization consistency among the designed probes was assessed using a pilot array. The validity of the genome-wide array was evaluated by successful identification of the well-characterized transcriptional responses to heat and salt stress. Next, the genomic transcriptional responses of *Synechocystis *to S deprivation were investigated. For photosynthetic organisms, including plants and microbes, S is an essential macronutrient present in proteins, lipids, electron transport components, and many cellular metabolites. It is critical for the association of metal ions to form protein iron-sulfur (Fe-S) clusters, which perform indispensable functions including electron transfer, redox sensing, gene regulation, catalysis and maintenance of protein structure. Due to the limited intracellular storage of S, a continuous supply of this nutrient from the environment, mainly in the form of sulfate (SO_4_^2-^) anion, is necessary for cell growth and development. Following its acquisition, sulfate is either used for the direct sulfatation of compounds or it is reduced and converted to cysteine and methionine. S limitation can occur in natural freshwater environments and strongly affect community composition. In the laboratory, it was found that S deprivation was correlated with increase in H_2 _production in both green alga *Chlamydomonas reinhardtii *[23-26] and cyanobacterium *Synechocystis *[9]. The genomic transcriptional response of green alga *Chlamydomonas reinhardtii *in response to S deprivation has been investigated using DNA microarray analysis to obtain important regulatory insights underlying the physiological changes [27,28]. The cyanobacterial genome-wide transcriptional responses to C, N and Pi deficiencies have been investigated [19,21,22,29], but no S acclimation study has been done.

In the present study, we used our microarray platform to explore the transcriptional response of the *Synechocystis *genome to S deprivation. Our results demonstrated that the oligonucleotide microarray platform is applicable to systematic investigation of the transcriptional activities of the *Synechocystis *genome under the conditions of ecological or biotechnological interest, such as nutrient limitation and H_2 _production. The expression profiling indicated that regulation of gene transcription in the S acclimation conditions was critical for *Synechocystis *to sense nutrient limitations and tune its metabolism for efficient utilization of available resources. For example, repression of certain S-related genes was paralleled by activation of genes involved in internal and external S scavenging. It remains to be determined if the two processes share a common regulatory mechanism(s).

## Results and discussion

### Experimental condition and microarray data processing

*Synechocystis *transcriptional response to S deprivation was studied by two-color comparative hybridization using genome-wide oligonucleotide microarrays that were designed and validated as described in detail [see Additional file 1]. In short, a cost-effective two-stage design strategy was developed. In the first stage, genomic DNA was hybridized to the pilot array to evaluate the specificity of and hybridization consistency among the designed probes. In the second stage, the genome-wide microarray (representing 3064 protein coding genes) was developed and validated by successful identification of well-studied transcriptional responses including heat and salt stress.

*Synechocystis *culture was grown in HEPES (4-(2-hydroxyethyl)-1-piperazineethanesulfonic acid) buffered BG-11 medium [30] to mid-exponential phase, at which point (designated as time 0, +S condition) SO_4_^2- ^was removed from the medium (MgSO_4 _replaced by MgCl_2 _of the same molarity). Following S removal and a wash in S-free medium, time-point samples were taken over a short-term period (1, 3, 6, 12, 24 hr) and a long-term period (48, 72 hr) in order to capture both "rapid" and prolonged transcriptional responses. RNA isolated from the time-0 sample was used as a common reference in time-course comparisons. For each time-point, 2 or 3 biological samples were examined. The scanned fluorescence intensity data were smoothed to remove undesirable local hybridization biases, and normalized using an ANOVA model [31] to remove systematic biases. Following the data reduction, differentially expressed genes were identified on the basis of passing a false discovery rate (FDR) cut-off applied to a two-class *t*-statistic. In addition to individual gene analysis, the gene set analysis [32] was used to identify gene sets which were strongly or moderately associated with the S deprivation response by assessing the significance of differential expression of pre-defined gene sets. The 78 gene sets were assembled based on functional classification of *Synechocystis *genes from *Cyanobase *[13], out of which 14 were first-level categories and 64 second-level categories. The gene sets and annotation are available [see Additional file 2].

### Global features of temporal response

**Figure 1:**
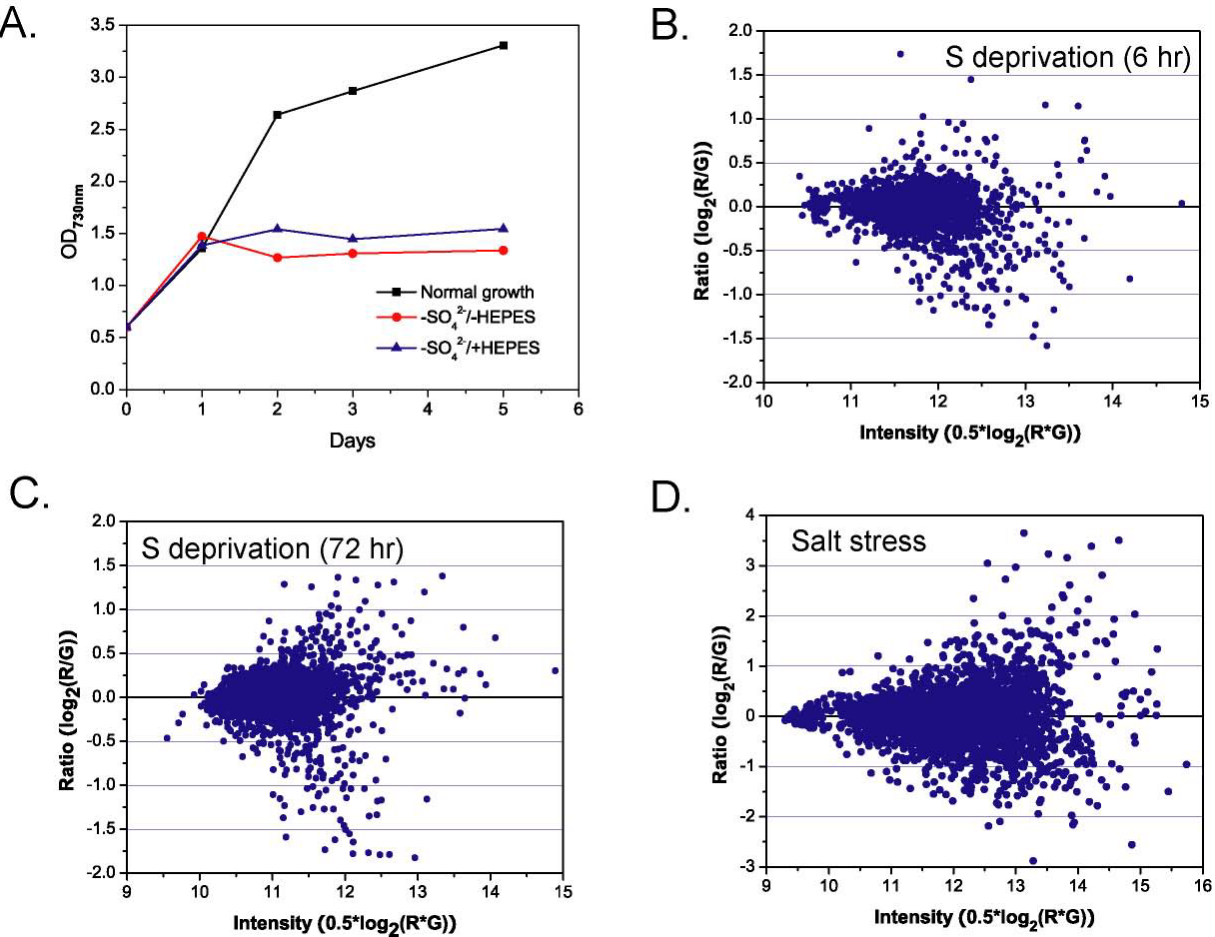
**Growth and genome-wide transcriptional effects of sulfur starvation**. **A**. Growth curves. S starvation, with or without HEPES buffering, was initiated at mid-exponential phase (designated as time 0). A normal growth control culture was examined in parallel. **B**. and **C**. Representative ratio-intensity plots showing the dispersion of transcript abundance ratios 6 hr (B) and 72 hr (C) after sulfate removal. **D**. A representative ratio-intensity plot for salt stress experiments which was conducted as described [see Additional file 1]. R: F_635median _intensity ("experiment"); G: F_532median _intensity ("control"). The fluorescence intensity data were pre-processed as described in *Methods*.

S deprivation had a slow-developing effect on cell growth and transcriptional activity of *Synechocystis*, as shown in Figure 1. Within 24 hr, S deprivation did not noticeably reduce *Synechocystis *growth rate compared to the normally growing control (Figure 1A). Consistent with a previous report [33], the growth was arrested after long-term deprivation (here defined as >24 hr). Only a few dozen genes demonstrated high magnitude changes in transcription (>1.5 fold) at each time-point. When panels B and C in Figure 1 are compared, it could be noted that the number of genes whose transcript levels were affected after 72 hr of starvation was greater than after 6 hr. At each time-point, the number of significantly down-regulated genes was roughly twice that of the up-regulated ones. The predominance of low-magnitude effects cannot be entirely attributed to the microarray platform and/or data processing, as we were able to detect more than 10-fold changes and hundreds of highly responsive genes in the salt stress treatment (Figure 1D).

### Transcriptional dynamics of significantly affected genes and gene sets

**Figure 2:**
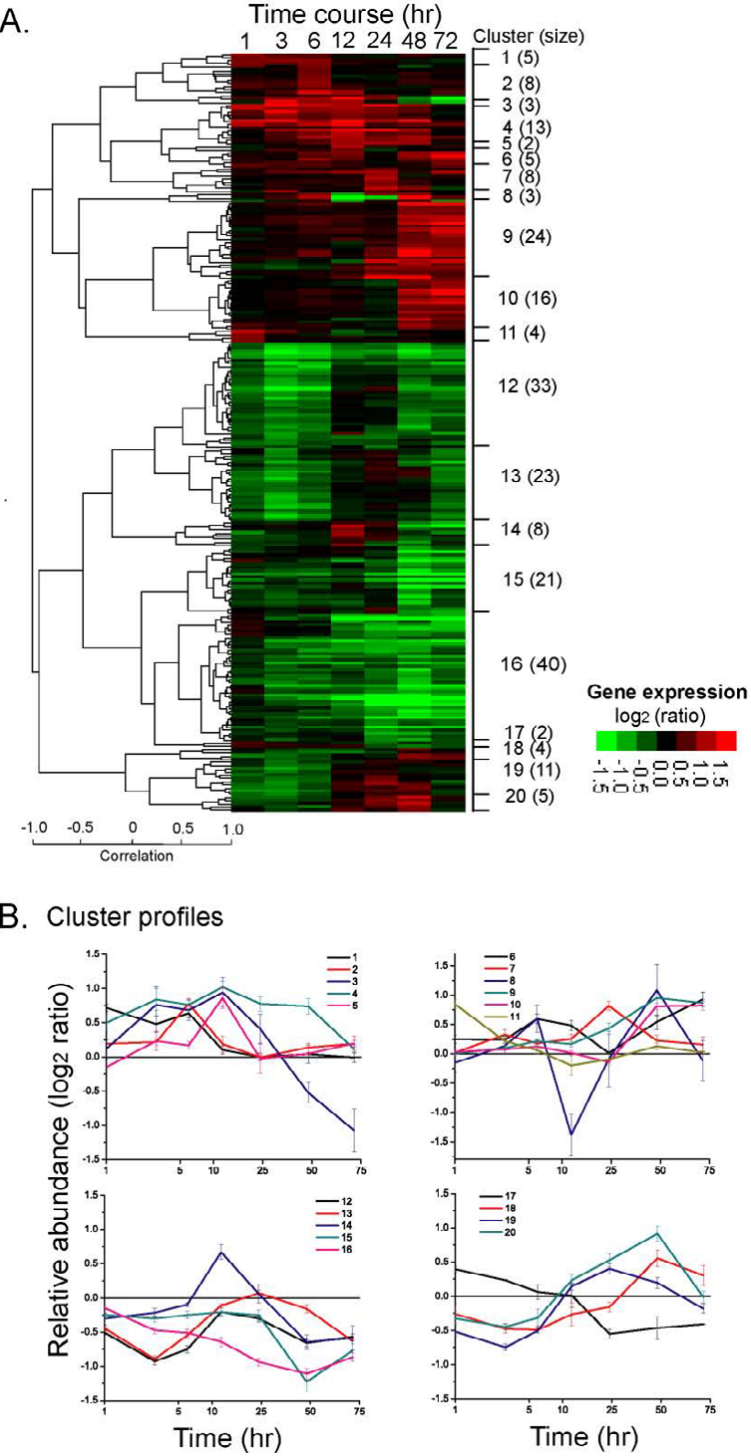
**Cluster analysis of temporal transcriptional response elicited by S withdrawal (-SO_4_^2-^/-HEPES)**. **A**. A heat map of a hierarchical cluster of genes significantly affected by S deprivation. A gene was deemed significantly affected if its transcript level deviated from a 0-hr level by at least 50% in at least 1 time point across the time course (FDR<0.01). Hierarchical clustering was performed by complete linkage algorithm using Pearson correlation as a similarity metric. The log_2 _(ratio) of each gene is represented according to the shown color scale. 20 clusters were delineated based on a correlation coefficient (*r*) cut-off of 0.50, shown to the right of the heat-map. Numbers given in parentheses represents the number of genes contained in each cluster. **B**. The transcriptional profile of each cluster. The data are represented as a mean ± s.e.m.

The transcriptional dynamics of significantly affected genes were examined in time courses following S withdrawal. The statistically significant genes were selected based on FDR cutoff of <1% for each time-point experiment (FDR was calculated using permutations [34]). From the list of genes satisfying the FDR criterion, average transcript abundance of 238 genes changed more than 1.5-fold in at least one time point were selected for subsequent analysis [see Additional file 3]. These genes were hierarchically clustered using Pearson correlation as a distance metric and displayed as a heat-map, as shown in Figure 2A. Twenty clusters could be delineated based on a correlation coefficient (*r*) cut-off of 0.5. Most of the clusters contained more than five genes. Figure 2B shows the transcriptional profile of each cluster, plotted as a mean ± s.e.m. A partial list of the differentially expressed genes encoding proteins with known functions, along with their transcriptional profile classifications, is given in Table 1.

**Table 1:** Selected significantly affected genes with known functions.

**Cluster**	**Selected constituent genes**
1	slr1933(*rfbC*), sll0398(*dgt*), slr1224(*malK*)
2	sll0336(*accD*), sll1129(*todF*), sll0535(*clpX*)
4	Phycobilisome degradation protein NblA (ssl0452 and ssl0453), sll1869(*cbaB*), Sulfate transport system (slr1452(*sbpA*), slr1453(*cysT*), slr1454(*cysW*), slr1455(*cysA*))
6	sll1165(*mutS*), slr1511(*fabH*), sll1076(*ziaA*)
7	sll0306(*rpoD*), slr0074(*ycf24*)
9	sll1459, sll1621, slr0143, slr1041, slr0537, sll0856(*rpoE*), slr0853(*rimI*) CO_2 _fixation slr0436(*ccmK*), Soluble electron carriers sll1245(*cytM*)
10	sll1958(*hisC*), slr1129(*rne*), slr0918(*pepM*)
11	ssl0546(*minE*), sll1635
12	sll1363(*ilvC*), slr1756(*glnA*), slr0623(*trxA*), slr1622(*ppa*), sll1070(*tktA*) ATP synthase (sll1321(*atp1*), sll1322(*atpI*), sll1324(*atpF*), sll1325(*atpD*), sll1326(*atpA*), slr1329(*atpB*), ssl2615(*atpH*)), CO_2 _fixation (sll1028(*ccmK*), slr0009(*rbcL*)), Cytochrome b_6/f _complex (sll1317(*petA*), slr0342(*petB*)), Translation (sll0517(*rbpA*), sll1098(*fus*)), Ribosomal proteins (sll1744 (*rpl1*), sll1746 (*rpl12*), sll1807 (*rpl24*), sll1808 (*rpl5*), sll1809 (*rps8*), sll1810 (*rpl6*), sll1813 (*rpl15*), ssl1426 (*rpl35*))
13	RNA polymerase subunits (sll1787(*rpoB*), sll1789(*rpoC2*), sll1818(*rpoA*)), Translation (sll1820(*truA*), sll1099(*tufA*), Ribosomal proteins (sll0767(*rpl20*), sll1745(*rpl10*), sll1799(*rpl3*), sll1801(*rpl23*), sll1802(*rpl2*), sll1803(*rpl22*), sll1804(*rpl3*), sll1805(*rpl16*), sll1806(*rpl14*), sll1812(*rps5*), sll1817(*rps11*), sll1819(*rpl17*), sll1821(*rpl13*), sll1822(*rps9*), ssl0601(*rps21*), ssl3432(*rps19*), ssl3436(*rpl29*))
14	Translation (ssr1480, ssl3441 (*infA*), Ribosomal protein ssr2799 (*rpl27*))
15	sll1184(*ho1*), slr0444(*aroA*), slr1176(*agp*), sll0807(*cfxE*), slr1545(*rpoE*), Cytochrome b_6/f _complex subunit smr0003(*petM*), Photosystem I (smr0005(*psaM*), ssl0563(*psaC*)), Photosystem II (sll1867(*psbA3*), sml0001(*psbI*), sml0002(*psbX*), sml0005(*psbK*), smr0001(*psbT*), smr0007(*psbL*), smr0008(*psbJ*), ssl2598(*psbH*)), Phycobilisome (sll1578(*cpcA*), slr2051(*apcG*))
16	sll1626(*lexA*), slr0165(*clpP*), sll0947(*lrtA*), CO_2 _fixation slr0012(*rbcS*), Cytochrome b_6/f _complex slr0343(*petD*), Photosystem I (sll0819(*psaF*), slr0737(*psaD*), slr1655(*psaL*), slr1834(*psaA*), slr1835(*psaB*), sml0008(*psaJ*)), Photosystem II (sll0258(*psbV*), sll0427(*psbO*), sll0849(*psbD*), sll0851(*psbC*), slr0906(*psbB*), slr0927(*psbD2*), slr1311(*psbA2*), smr0006(*psbF*), ssr3451(*psbE*)), Phycobilisome (sll1577(*cpcB*), sll1579(*cpcC*), slr0335(*apcE*), slr1459(*apcF*), slr1986(*apcB*), slr2067(*apcA*), ssr3383(*apcC*)), Soluble electron carriers (sll0199(*petE*), ssl0020(*petF*))
18	slr1562(*grxC*), sll0018(*cbbA*)
19	slr0164(*clpP*), Cytochrome b_6/f _complex slr1185(*petC*), slr1828(*petF*), Ribosomal proteins (sll1816(*rps13*), slr1984(*rps1*), sml0006(*rpl36*), ssl3437(*rps17*), ssl3445(*rpl31*), ssr0482(*rps16*))
20	slr2075(*groES*), Ribosomal proteins (sll1824, slr0628(*rps14*), ssl2233(*rps20*))

The differentially expressed genes in *Synechocystis *sulfate deprivation time course were identified and clustered according to Figure 2. The complete list, with annotations (gene descriptions and functional categories) and mean fold change for each time-point comparison, is available [see Additional file 3].

The cluster analysis revealed the structure and dynamics of the transcriptional data obtained in the study. Overall, the profiles of up-regulated genes (91 genes broadly grouped in clusters 1 to 11) and down-regulated genes (147 genes in clusters 12 through 20) were highly anti-correlated with a Pearson correlation coefficient of -0.99. Clusters 1 to 5 showed an early induction with average transcriptional changes peaking before the 24-hr time point. Cluster 3, containing three genes encoding for proteins with hypothetical functions, showed a late repression. Genes belonging to cluster 4, which included genes encoding for phycobilisome (PBS) degradation (*nblA*) and sulfate transport system (*sbpA-cysTWA*), were consistently up-regulated until 72 hr after S withdrawal. Clusters 6 to 10 contained genes that were significantly induced at a late stage (after 24 hr); 34 out of a total of 51 genes encoding for proteins with hypothetical or unknown functions could be found in those clusters. Cluster 11 contained 2 genes encoding for transposon-related functions, showing an immediate up-regulation. Clusters 12 to 16 showed overall down-regulation in transcript abundances. The lowest transcript levels for clusters 12 and 13, enriched for genes encoding ATP synthase, CO_2 _fixation and cytochrome b_6/f _complex, was reached at 3-hr time point. However, for clusters 15 and 16, which contained all of the genes responsible for light harvesting and photosynthesis (PBS, photosystems I and II) and most of the genes encoding for transcription and translation machineries, the largest repression occurred 48 hr following S removal. Cluster 14, which contained 3 genes encoding components of the translational machinery and 4 encoding hypothetical and unknown protein, showed a single-peak transitional profile (*down-up-down*). Cluster 17 exhibited initial up-regulation followed by late down-regulation. Clusters 18 to 20 showed a dynamic profile reciprocal to that of cluster 17: an initial down-regulation followed by steady up-regulation in later time points. 12 out of the 16 genes contained in clusters 19 and 20 encoded proteins involved in protein synthesis and folding (ribosomal subunits, chaperone, ClpP protease) as well as components of the protein/peptide secretion machinery. In sum, the clustered transcriptional profiles indicated a three-stage temporal dynamic of genome-wide transcriptional regulation: early (<12 hr), transitional (12~24 hr) and late (>24 hr) stages. The genome-wide transcriptional activity may underpin the observed growth phenotype, which exhibited at least a two-stage response to S starvation: 1–2 normal doublings followed by the growth arrest.

The cluster analysis also revealed co-expression, and, potentially, co-regulation. First, genes in the same operon were found to be co-regulated (e.g., *nblA *(*ssl0452-3*), *sbpA-cysTWA *(*slr1452-5*), hypothetical (*slr0146-9*), *atpIHGFDACBE *(*sll1321-9*), *psaAB *(*slr1834-5*), *psbFLJ *(*smr0006-8*), *apcABC *(*slr2067-slr1986-ssr3383*)). Most of the genes encoding for ribosomal protein subunits and sharing the same cluster profile were distributed continuously on the *Synechocystis *chromosome. Second, genes encoding components of multi-protein complexes, which are scattered along the *Synechocystis *chromosome, were also co-expressed. For example, *psbD *(*sll0849*) and *psbD2 *(*slr0927*) were clustered together (cluster 16), both encoding for photosystems (PS) II reaction center D2 protein; *rpoA *(*sll1818*) shared the same cluster with *rpoB *(*slr1787*) and *rpoC2 *(*sll1789*), all encoding for core subunits for RNA polymerase. However, several duplicated genes indicated disparate transcriptional profiles. The *psbA2 *(*slr1311*) and *psbA3 *(*sll1867*) genes, encoding form II and III of PS II reaction center D1 polypeptide, were found to be significantly down-regulated, while *psbA1 *(*slr1181*), which is thought to be a cryptic gene [35], showed no change in its transcript abundances during the time course. This pattern of regulation was also noted for high light [16] and salt stresses [17]. Other disparately active genes included those encoding the proteins sensing CO_2 _concentration CcmK (*sll1028 *and *slr0436*), ferredoxin PetF (*slr1828 *and *ssl0020*), ATP-dependent protease ClpP (*slr0164 *and *slr0165*), and a group 2 sigma factor RpoE (*sll0856 *and *slr1545*). The distinct transcriptional activities of those genes may indicate their complementary roles in mediating cellular stress responses.

In addition to individual genes, we also identified groups of genes that were significantly affected in the experiment. The top-ranking significantly up-regulated or down-regulated sets of genes are listed in Table S2 [see Additional file 4]. Consistent with the analysis of differentially expressed genes, the sets of photosynthetic and respiration genes (enriched in clusters 12, 15 and 16) as well as protein synthesis genes (enriched in clusters 12 and 13) were significantly down-regulated. The sets of genes whose products are involved in protein and peptide secretion, phosphorus and energy metabolism (pentose phosphate pathway, sugars) were also among the most down-regulated groups. The sets of genes involved in RNA metabolism, including transcription and degradation, were down-regulated by 12 hr of S starvation. However by 24 hr, at least some genes involved in RNA degradation appeared to be significantly up-regulated, including the ribonuclease E encoding gene *rne*, in cluster 10. Up-regulated gene sets included respiratory terminal oxidases, transport and binding proteins, cell envelope, membranes, lipoproteins, and porins, fatty acid, phospholipid and sterol metabolism. Of note, many sets of genes encoding for biosynthesis of cofactors, prosthetic groups and carriers (folic acid, cobalamin, heme, phycobilin and porphyrin, riboflavin, molybdopterin) were also significantly up-regulated. This might be relevant since these cofactors participate in important primary as well as secondary metabolic pathways, e.g., pigment synthesis, metal homestasis, and electron transfer.

### Verification of microarray data by quantitative RT-PCR

The microarray data were partly verified by quantitative real-time PCR following reverse transcription of total RNA isolated from independent biological replicas of time point samples. While 16S rRNA is routinely used as an endogenous control for cell number, it may not be an ideal internal standard under conditions of variable growth [36]. Therefore in addition to 16S rRNA, we used *trpA *and *lysC *as an internal reference. The *trpA *gene was previously shown to be non-responsive to light or to chemical treatments [37]. More importantly, both *trpA *and *lysC *were not differentially affected in our experimental conditions (data not shown). We confirmed that during early S deprivation (1, 3, 6 hr), the abundance of *cpcA *(phycocyanin α subunit) and *psbA2 *(PS II D1 protein) transcripts was significantly decreased, while that of *nblA2 *(*ssl0453*, PBS degradation protein NblA) and *cysW *(sulfate transport system permease protein) was consistently increased (Table 2A). The three internal references produced qualitatively similar results. We observed that the quantitative PCR technique yielded relatively larger differences in transcript abundances than those estimated from microarray ratios. It has also been observed in other studies that the quantitative PCR values are, in general, much higher than microarray data, whether based on oligonucleotide or cDNA microarray platform [38,39]. The somewhat larger disparity between microarray signals and the quantitative PCR observed in this study was likely due to the fact that, in the reported microarray experiment, fluorescent intensities were not corrected for background noise: surprisingly, application of available background models, suitable for microarrays with double stranded DNA probes, resulted in a dramatic deterioration of the statistical properties of the data. At the same time, quantitative PCR can have close-to-100% amplification efficiency at optimal conditions. Though the magnitudes of microarray data are smaller, it can sensitively discriminate the direction of transcriptional changes (up-regulation or down-regulation) for a large number of genes simultaneously.

**Table 2:** Quantitative real-time RT-PCR analysis of selected transcripts.

A									
**Gene**	**1 hr**			**3 hr**			**6 hr**		

	*rRNA*	*trpA*	*lysC*	*rRNA*	*trpA*	*lysC*	*rRNA*	*trpA*	*lysC*
*cpcA*	-1.95 ± 0.23	-2.79 ± 0.01	-2.35 ± 0.01	-1.66 ± 0.11	-2.28 ± 0.17	-2.23 ± 0.06	-1.40 ± 0.21	-1.38 ± 0.02	-1.95 ± 0.04
*psbA2*	-3.08 ± 0.10	-2.97 ± 0.02	-2.52 ± 0.02	-2.03 ± 0.04	-2.07 ± 0.17	-2.01 ± 0.05	-0.94 ± 0.17	-0.45 ± 0.02	-1.02 ± 0.04
*nblA2*	0.12 ± 0.10	1.20 ± 0.20	1.14 ± 0.15	3.77 ± 0.03	-0.88 ± 0.09	0.25 ± 0.23	2.98 ± 0.09	n.d.	1.99 ± 0.21
*cysW*	0.80 ± 0.12	n.d.	n.d.	6.06 ± 0.10	1.41 ± 0.13	2.54 ± 0.25	4.56 ± 0.38	n.d.	3.57 ± 0.43

*B*									

**Gene**	**1 hr**			**3 hr**			**6 hr**		

*hoxH*	0.65 ± 0.06			0.83 ± 0.07			0.06 ± 0.05		
*hoxE*	0.52 ± 0.03			0.72 ± 0.05			-0.14 ± 0.06		
*hypA1*	0.22 ± 0.01			0.13 ± 0.06			-0.21 ± 0.07		
*hypB1*	-0.01 ± 0.09			-0.31 ± 0.07			-0.86 ± 0.06		
*lexA*	0.52 ± 0.08			-0.4 ± 0.09			-0.98 ± 0.08		

A. Transcript abundances of significantly induced *nblA2*(ssl0453), *cysW*(slr1454) and repressed *cpcA*(sll1578), *psbA2*(slr1311) genes, relative to stable RNA, *trpA*, or *lysC*, as endogenous references. B. Measurements of transcript abundances of hydrogenase-related genes using *lysC *as an endogenous reference. Mid-exponential cells were harvested and fixed in phenol-ethanol stop solution before sulfate withdrawal (time 0) and 1, 3, 6 hr after sulfate withdrawal. Total RNA was isolated and reverse transcribed. The relative abundances of each transcript were measured by SYBR Green real-time PCR and analyzed according to the comparative *C*_*t *_method. The data are presented as transcript abundances, normalized to a reference mRNA, in a time-point sample *relative *to control (time 0) on the log_2 _basis. Errors are the pooled s.e.m. (n = 3). "n.d." – not determined.

### Control experiments indicated that sulfate deprivation induced specific transcriptional changes

Because the medium pH may affect H_2 _production [9,23], the S deprivation experiment was also performed with HEPES buffering. Both the growth curve (Figure 2A) and transcriptional profile (Figure S1) [see Additional file 4] were comparable with the HEPES-free condition. Out of the top 100 significantly affected genes, 93 were common between the two S deprivation conditions. Another control experiment was done to verify that the transcriptional regulation was indeed due to S deprivation and not a growth stage-dependent artifact. As shown in Figure S1 [see Additional file 4], the growth stage time-course profile was significantly different from the profile of S deprivation conditions. Out of the top 100 significant genes, about half of *down-regulated *genes in S deprivation conditions were *up-regulated *in normal growth stage transition, including many PSI genes. However, both PSI and PSII genes were significantly down-regulated in late stationary phase (data not shown). Typically the sulfate transporter *sbpA-cysTWA *operon was not up-regulated during normal growth control. Many differentially expressed individual genes (Figure S1) and gene sets (Table S2 B) [see Additional file 4], including up-regulation of detoxification genes and simultaneous down-regulation of genes for N (glutamate family) and S (serine family) assimilation, represented known transcriptional features of cells transitioning from exponential to stationary phase of growth [40]. These results suggested that the transcriptional regulation following S deprivation was indeed attributable in large part to sulfate availability and not a general response to slowed growth.

### S starvation induced general and specific responses to macronutrient deficiencies

The S deprivation microarray data identified both the general and specific responses to macronutrient deficiencies [29,41,42]. The specific responses result from a particular nutrient limitation, whereas the general, or common, responses occur under any starvation condition such as C, N or Pi deprivation. Upon S deprivation, the high-affinity periplasmic sulfate transport system, encoded by *sbpA-cysTWA *operon in *Synechocystis*, was rapidly and significantly induced (>2 fold), and stayed induced until 72-hr after S withdrawal (Cluster 4 in Figure 2). The physiological roles of the proteins encoded by these genes in sulfate acquisition have been elucidated in *Synechococcus *sp. strain PCC7942 [41], another extensively studied freshwater cyanobacterium. Their homologs in *Synechocystis *may have a similar function. SbpA (sulfate binding protein) is a polypeptide that binds to sulfate. The binding causes a conformational change in the protein which results in trapping of a sulfate substrate molecule. The protein ligand complex then interacts with two hydrophobic proteins (CysTW) which are thought to span the cytoplasmic membrane and form a pore. This interaction results in the release of the substrate. It then traverses the pore and enters the cell against a concentration gradient using energy derived from ATP hydrolysis which is a function of the nucleotide binding protein encoded by *cysA*. Though sulfate acquisition genes were significantly induced, some S assimilation pathway genes, including sulfate adenylytransferase (*met3*), adenylylsulfate kinase (*cysC*), phosphoadenosine phosphosulfate reductase (*cysH*), D-3-phosphoglycerate dehydrogenase (*serA*), serine hydroxymethyltransferase (*glyA*) and cystein synthase (*cysM*, *cysK*), were not identified in our analysis as being significantly affected. The responses mentioned above are specifically related to S limitation/starvation. The common response of cyanobacteria upon nutrient deficiencies typically includes shutting down photosynthesis and degrading their PBS, the specific light harvesting complexes for PS II. As analyzed above, the photosynthesis and respiration genes, including those encoding the five major macromolecular complexes localized on the thylakoid membrane, PBS, PS II, cytochrom b_6/f _complex, PS I and ATP synthase, were consistently down-regulated during the experimental time course. PBS structural genes, including genes encoding core (*apcABC, apcF*), rod (*cpcBAC*) and linker (*apcG*, *apcE, cpcC*) polypeptides, were significantly down-regulated. PBS, which can constitute up to half of the total cellular protein [43], is known to be rapidly and progressively degraded during nutrient-limited growth. The PBS degradation was thought to minimize the absorption of excess excitation energy under stressful conditions [44]. However, another possibility is that it can be scavenged as a source of S-containing amino acids, although PBS is thought to be a poor S-source [41]. The PBS degradation is triggered by a small polypeptide encoded by *nblA *(non-bleaching) genes (*ssl0452 *and *ssl0453 *in *Synechocystis*), which were significantly up-regulated in our microarray experiments (cluster 4 in Figure 2) and confirmed by quantitative reverse-transcription PCR (see above). The opposite transcriptional regulation of the PBS structural genes and PBS degradation protein genes suggests that *Synechocystis *down-regulates PBS abundance and/or activity for light harvest and energy transfer at the transcriptional level, though this down-regulation may not be causing dramatic bleaching in the process (known as *chlorosis*) [33]. The declined PBS transcription was correlated with the down-regulation of the downstream energy transfer components: PS II, cytochrom b_6/f _complex, PS I and ATP synthase (Table 1). Furthermore, key CO_2 _fixation genes, corresponding to the ribulose bisphosphate carboxylase (*rbcL*, *rbcS*) and the CO_2 _concentrating mechanism protein CcmK (*sll1028*), were down-regulated; soluble electron carrier genes (*petE*, *petF*), except *cytM*, which encodes cytochrome cM, were all consistently repressed upon S removal. Notably, no genes in respiratory terminal oxidase and NADH dehydrogenase sub-categories were identified as down-regulated, from both the individual gene analysis (Table 1) and gene set analysis (Table S2) [see Additional file 4]. It may indicate that cytochrome oxidase-mediated respiration activity was not significantly repressed on a transcriptional level during S acclimation.

### S deprivation elicited transcriptional responses associated with general growth arrest and stress

Other components of the transcriptional response were associated with general growth arrest and stress. First, down-regulation of both transcription and translation machineries was observed. RNA polymerase core subunits genes (*rpoA*, *rpoB*, *rpoC2*) were significantly repressed. The ribosomal protein subunit genes and genes encoding the initiation factor IF-1 (*infA*), and two elongation factors (*fus *and *tufS*) were significantly down-regulated. Many of these significantly repressed ribosomal protein subunits genes are clustered on the *Synechocystis *chromosome (from *sll1799 *to *sll1822*). The transcription of the principle sigma factor SigA, encoded by *rpoD *(*sll0306*, cluster 7), was significantly induced at 24-hr time-point, and the ECF-type (group 3) sigma-E factor, encoded by *rpoE *(*sll0856*, cluster 9), showed pronounced late induction. They may be involved in the transcriptional modulation during the transition stage (12–24 hr). SigE is also involved in the response to N deprivation as well as in positive regulation of sugar catabolism [20]. Second, transcriptional adjustment of genes involved in macromolecular biosynthesis and energy metabolism was observed. Transcriptional regulation of several genes involved in N assimilation, including *glnA *(encoding type I glutamine synthetase, *repressed*), *sll1911 *and *sll1515 *(both encoding for glutamine synthetase inactivating factor IF7, induced significantly at both early and late stage), may indicate the slowing of ammonium assimilation under S limitation. Similarly, *ppa *(soluble inorganic pyrophosphatase), a key Pi starvation induced gene [45], was down-regulated. The repression of pyrophosphatase activity, however, may suggest accumulation of polyphosphate during S deprivation. S deprivation induced a decline in photosynthetic electron transfer, concurrent with down-regulation of PBS and photosynthesis, which were exemplified by reduced abundances of *trxA *(thioredoxin) and *grxC *(glutaredoxin). In addition, three energy metabolism genes, *cbbA *(fructose-bisphosphate aldolase), *cfxE *(pentose-5-phosphate-3-epimerase) and *tktA *(transketolase), were repressed. All three gene activities lead to production of glyceraldehydes 3-phosphate, through glycolysis (*cbbA*) or non-oxidative branch of pentose phosphate pathways (*cfxE *and *tktA*). The effect of this regulation may be to lower the sugar catabolism and preserve carbohydrate storage in the form of glycogen. However, genes for fatty acid and lipid biosynthesis, *accD *and *fabH*, were significantly up-regulated, which is interesting with respect to the carbon storage and H_2 _production hypothesis [5,8,9]. Third, stress protection responses were observed. The stress response involved genes encoding chaperon and proteolysis functions (*groES*, *clpX, clpP, rbcX*), preprotein translocase (*secE*, *secY*), a pigment biosynthesis pathway gene (*ho1*), a stationary phase survival protein SurE homolog (*sll1459*), ribonuclease E (*rne*), light-repressed protein A homolog (*lrtA*), antioxidant protein (*slr1198*), oxidative stress protection AhpC/TSA family protein (*sll1621*), and two-component response regulator genes (*sll0789 *and *slr1041*). Additionally, control of the cell cycle (*minE*), nucleotide synthesis (*dgt*, *sll1635*), DNA protection (*ruvA*, *mutS*), and modulation of cell envelop permeability (*slr1841*, *slr0143*, *rfbC*) were affected by S stress.

Of note, a large number of genes regulated by S availability encode hypothetical proteins or proteins of unknown function [see Additional file 3]. Interestingly, about 30% of those genes were significantly up-regulated during the late stage of S starvation (clusters 9 and 10). Their involvement, if any, in S acclimation and/or utilization remains to be elucidated.

Overall, our genome-wide analysis presented a broad view of transcriptional changes accompanying sulfur starvation in cyanobacteria. Some strategies unveiled here are also "employed" by cyanobacteria to cope with C, N and Pi deficiencies [19-21,45]. However, to fully understand how cells sense, transmit and integrate information about S status, it may be necessary to study various mutants affecting S acclimation, including: (1) a mutant of the *sll0640 *gene, whose *Chlamydomonas reinhardtii *homologue *sac1 *(sulfur acclimation) is a putative sensor of S levels in the environment and is required for survival of S-deprived *Chlamydomonas *cells in the light [46]; (2) mutants of *dspA *(also known as *hik33*) [47] and *sll0396*, whose *Synechococcus *orthologues, *nblS *and *nblR*, respectively, encode the components of the histindine kinase-response regulator (Hik-Res) two-component system NblS-NblR [48], which was shown to be critical for acclimation of *Synechococcus *to high-light and nutrient limitation conditions [29]. It is possible, however, that other two-component signalling systems mediate S acclimation in *Synechocystis*, including those that are regulated by *sll0789 *of the OmpR subfamily and *slr1041 *of the PatA subfamily, which were significantly up-regulated in our experimental condition; (3) mutants of global regulators of C, N and Pi metabolism, such as NtcA [20] and NdhR [19], which mediate a "cross-talk" between nutrient-specific responses in the cell [19,29].

It might also be informative to compare the S acclimation response of cyanobacteria with eukaryotic oxygenic photosynthetic organisms and heterotrophic eubacteria. In green alga *Chlamydomonas reinhardtii*, S deprivation leads to increased accumulation of arylsulfatase, which releases sulfate anions from esterified organic sulfates, allowing cells to access new sulfur stores [49]. In the opportunistic pathogen *Pseudomonas aeruginosa*, S deprivation induces genes encoding sulfatases and sulfonatases for desulfurizing a range of organic sulfur compounds such as sulfate esters and sulfonates [50]. In contrast, the sulfate starvation response in *E. coli *is dominated by increased expression of the S assimilation cysteine biosynthetic pathway, and propagated into methionine metabolism, synthesis of Fe-S clusters, and oxidative stress [51]. These differences, to some extent, may be associated with the special cellular component that only cyanobacteria have – PBS. On one hand, PBS degradation may directly contribute to nutrient scavenging (mainly for N, moderately for S, none for Pi) because it constitutes up to 50% of the total cellular protein. On the other hand, the transcriptional shut down of PBS biosynthesis, accompanied by reduced photosynthetic activity, allows nutrient limited cells to survive for relatively long periods of time under stress conditions via a "*stand-by*" mode of energy metabolism.

### Relevance to biohydrogen production

As a secondary metabolic process of significant biotechnological interest, photobiological H_2 _production from water by cyanobacteria depends on coordination between photosynthetic electron transport and the catalytic activity of the hydrogenase. The optimal conditions for H_2 _production should direct more electrons to hydrogenase than other competing pathways (O_2_, nitrate, Calvin-cycle, etc.) [52-55]. At the same time, anaerobic conditions are necessary to induce hydrogenase expression and/or activity [56]. We observed that S deprivation significantly increased photoautotrophic H_2 _evolution by *Synechocystis*, compared with +S controls (Figure S2) [see Additional file 4]. The microarray expression profiling suggested similar underlying principles for H_2 _production as observed in the green alga *Chlamydomonas reinhardtii *[2,26,55,57,58]. S deprivation leads to inactivation of PS II which performs water splitting. Oxygen evolution ceases and it is depleted by respiration, as it was observed that PS II genes were significantly repressed but genes encoding for NADH dehydrogenase and respiratory terminal oxidase were not. This process leads to anaerobiosis, which in turn could induce hydrogenase activity. S starvation inhibits CO_2 _fixation and thus removes another important electron sink. Furthermore, this process likely leads to decreased sugar catabolism and the accumulation of carbohydrates, which is important for sustained H_2 _production in the long run. Previous work [59] proposed that S limitation triggers H_2 _production by causing a lack of the D1 protein (32-kDa reaction center protein) which is essential for PS II and needs to be constantly replaced. The microarray data suggested that H_2 _evolution was accompanied by a complex and dynamic transcriptional process. However, the question remains whether, and to what extent, the observed complexity of transcriptional regulation is essential for renewable, i.e., without the loss of viability, bioproduction of H_2 _by cyanobacteria.

The mRNA levels of genes encoding activities directly involved in H_2 _evolution were specifically focused on by quantitative RT-PCR. *Synechocystis *uses heteropentameric [NiFe] hydrogenases (HoxEFUYH) and maturation accessory proteins (*hyp *genes) for H_2 _production. None of those genes was identified as significantly differentially affected in microarray experiments. Moreover their known transcriptional activator LexA [60] was down-regulated in the conditions we tested (Table 1). The qRT-PCR assay confirmed that the transcript levels of most of the hydrogenase genes were not significantly increased (Table 2B). This indicates that hydrogenase levels were not limiting at the transcript level. However, *hoxE *and *hoxH *were induced more than 1.5 fold at 1-hr and 3-hr time points as measured by qRT-PCR. There may be other factors involved in the regulation of the *hox *operon transcription besides LexA. For instance, recently an AbrB-like protein was identified as a positive regulator of expression of the bidirectional hydrogenase in *Synechocystis *[61].

However, cells only survive for a limited period of time in S-deprived medium and will eventually die. Such a shortcoming may be overcome by designing systems that mimic S deprivation for H_2 _production. For example, an inducible *nblA *gene expression system could be constructed under the control of promoters whose activity can be modulated by nonessential micronutrient inducers, e.g., *petE *promoter by copper [62], or by environmental parameters, e.g., *groES-groEL *promoter by heat shock. The cyclic induction and repression of *nblA *transcription may lead to repeated PBS degradation and synthesis, which in turn cyclically inactivates and activates PS II. An inducible PS II gene expression system can potentially generate repeated cycles of carbon storage and H_2 _production, allowing for scalable and sustainable H_2 _production. A similar idea has been explored in *Chlamydomonas reinhardtii *for triggering H_2 _production by means other than macronutrient limitation [63].

## Conclusion

Here we reported the study of transcriptional activity of the *Synechocystis *genome in response to S deprivation by using a newly designed microarray of more than 3,000 protein coding genes of *Synechocystis *sp. PCC 6803. The genome-wide expression profiling indicated that S deprivation induced relatively weak, but significant, transcriptional changes. A complex transcriptional change during S acclimation was described in terms of general and specific responses characteristic of macronutrient deficiencies. A large portion of significantly affected genes were the genes of unknown function, highlighting gaps in our understanding of the nature of nutrient adaptation.

## Methods

### Probe design and microarray production

The 70-nt oligomers were designed using ArrayOligoSelector [64] as detailed in the text. The oligos were synthesized by Invitrogen Corp. (Carlsbad, CA, USA), resuspended in 5 μl of 3 × SSC to a final concentration of 66.7 pmol/μl and spotted onto poly-L-lysine coated microscopic glass slides using OmniGrid Microarray printer (GeneMachines, San Carlos, CA, USA), as described previously [65]. All oligo sequences are provided [see Additional file 5].

### Sulfate deprivation experiment

*Synechocystis *sp. PCC 6803 was grown photoautotrophically in BG-11 medium supplemented with 8 mM NaHCO_3 _and buffered with 10 mM HEPES (pH 7.4) [9]. The cells were grown in 250 ml flasks at 32°C under a light intensity of 25 μmol photons m^-2 ^s^-1^. Cultures were bubbled with sterile air containing 1% (v/v) CO_2_. Log phase cells (OD_730 nm _= 0.6) were harvested by centrifugation (2000×g for 12 min) washed once and then re-suspended in sulfate-free media (MgSO_4 _replaced by MgCl_2 _of the same molarity). In addition, all S-containing trace metals in BG-11 were replaced by non-S containing metals. Cells were harvested and fixed for microarray analysis by adding 10% (v/v) ice-cold 5% phenol in ethanol stop solution at the following time points: before S-depravation (time 0), 1, 3, 6, 12, 24, 48 and 72 hr after S-depravation. S deprivation with HEPES buffering control experiment was performed as described above, except that HEPES buffer was used upon sulfate removal. Bacterial samples for a time course were taken at time 0, 12 and 24 hrs after sulfate withdrawal. Growth stage control experiment was done in parallel with S deprivation experiments. Samples were taken at 0, 1, 2.5, 4, 7, 11 and 48 hr after OD_730 nm _reached 0.60. All the experiments were done in biological replicates.

### Total RNA preparation and cDNA fluorescent labelling

The bacterial samples were fixed by a stop solution (see above) and centrifuged at 4°C. The cell pellets were snap-frozen in liquid nitrogen before storing at -80°C until required. Total RNA was isolated using a hot phenol-chloroform method [66] with 1×TE sucrose buffer (10 mM Tris, 1 mM EDTA, 0.5 M sucrose, pH 8) at 50°C instead of using TE buffer (10 mM Tris, 1 mM EDTA, pH 8) at 25°C. Residual DNA was removed by DNase I digestion. Direct fluorescent labelling was done by reverse transcription as described previously [66].

### Microarray hybridization, washing and scanning conditions

Printed microarray slides were post-processed as described previously [65]. Microarray hybridization and washing conditions were based on *E. coli *cDNA microarray protocols [66] with some modifications. To reduce cross-hybridization, a pre-hybridization step was performed for *Synechocystis *slides as follows: 45 ml pre-hybridization solution (containing 4.5 ml of 20× SSC, 1.1 ml of HEPES (pH 7), 250 μl of salmon sperm DNA and 250 μl of BSA) was preheated to 65°C in a water bath. Then microarray slides were incubated vertically in the preheated solution for 45 min at 65°C. The slides were washed 3 times in sterile dH_2_O and were spin-dried for 2 min at 600 rpm. The slides were then immediately used for hybridization. The hybridization mixture (total volume 21 μl) contained on-column cleaned Cy3-dUTP and Cy5-dUTP labelled samples, 3 μl of 20×SSC, 0.5 μl of 1 M HEPES buffer (pH 7), 2 μl of BSA and 2 μl of salmon sperm DNA as blocking agent and 0.75 μl of 5% SDS. The hybridizations were carried out under a 20×20 flat cover-slip in hybridization chambers (Monterey Industries, Richmond, CA, USA) submerged in a 65°C water bath for 5 to 6 hours. Slides were washed sequentially in each of the following solutions: Chamber 1: 330 ml of dH_2_O, 15 ml of 20× SSC, 10 ml of 10% SDS; Chamber 2: 350 ml of dH_2_O and 1 ml of 20× SSC; and Chamber 3: 350 ml of dH_2_O and 0.5 ml of 20× SSC. After the washes, the slides were air dried by centrifugation for 3 min at 600 rpm and scanned with an Axon GenePix 4000 B laser scanner (Molecular Devices, Sunnyvale, CA, USA) at the resolution of 10 μm per pixel. The resulting 16-bit TIFF images were analyzed by using the software GenePix Pro 4.0 (Molecular Devices, Sunnyvale, CA, USA).

### Microarray data analysis

For RNA hybridization experiments, the original fluorescence intensity data were pre-processed using *lowess *smoothing and an ANOVA linear model [31]. The normalized Cy-5/Cy-3 log_2_(ratio) values were taken to reflect the relative gene expression level changes. The statistically significant genes were identified by using SAM package [34] at less than 1% FDR at the 90^th ^percentile for each time-point. Genes differentially expressed throughout the time-course were selected as described in the text. Hierarchical clustering analysis was performed in CLUSTER and visualized in TREEVIEW [67]. The *Synechocystis *gene-sets were assembled according to *Cyanobase *database [13,68], and analyzed using a modified gene set analysis method [32]. It was shown that this gene set analysis method is more powerful than other gene set enrichment analysis methods [69,70]. The free Microsoft Excel Add-in, downloaded from [71], was used to perform both SAM individual gene and gene set analyses.

### Quantitative reverse-transcription – PCR

Relative abundances of several transcripts (Table S2) [see Additional file 4] were independently verified by SYBR Green real-time PCR according to the manufacturer's protocol (Applied Biosystems, Foster City, CA, USA). Bulk cDNA samples were synthesized from total RNA using Taqman reverse transcription reagents (Applied Biosystems, Foster City, CA, USA) and random hexamers as primers. The gene-specific primers were designed using Primer3 software [72] according to the real-time PCR guidelines, and listed in Table S2. The relative abundance of each transcript (normalized to internal control genes 16S rRNA, *trpA*, or *lysC*) with respect to the control sample was measured in triplicates and calculated according to the comparative *C*_*t *_method [73].

### Hydrogen evolution measurement

Seven ml cell samples were incubated in long cylindrical tubes with no head space under the same growth condition as that of S deprivation microarray experiment. The photoautotrophic incubation was allowed to proceed for a period of at least 24 hr before H_2 _was measured. Following incubation, the samples were centrifuged before H_2 _content of the supernatant was analyzed using a membrane inlet mass spectrophotometer (Bay Instruments, Easton, MD, USA) which has been shown effective in measuring dissolved gases in water [74] and cell culture samples [75]. The H_2 _content of the cell sample was calculated based on air equilibrated deionized water as a blank control. Hydrogen production was normalized by OD_730 nm_. The H_2 _evolution from a parallel +S control culture was also measured. The measurements were performed in technical triplicates.

### Microarray data availability

The microarray data are accessible online through NCBI Gene Expression Omnibus (GEO) [76] series accession number GSE11970.

## Competing interests

The authors declare that they have no competing interests.

## Authors' contributions

ZZ carried out the sulfur deprivation microarray and quantitative RT-PCR experiments, analyzed microarray datasets and drafted the manuscript. NDP designed and validated the oligonucleotide microarray platform. KNP carried out bacteriological experiments and hydrogen measurements and edited the paper. JBC designed the study and co-wrote the paper. AK designed and coordinated the study, performed microarray data analysis and co-wrote the manuscript. All authors read and approved the final manuscript.

## Supplementary Material

Additional File 1 microarray_construction. This supplementary text describes the oligonucleotide probe design and microarray construction and validation. Click here for file

Additional File 2 genesets. This file contains gene set annotations assembled from *Cyanobase*. Click here for file

Additional File 3 s_deprivation_genes. A complete list of genes significantly affected by S deprivation. A statistical significance threshold was set at 1% false discovery rate (FDR). Top ranking differentially expressed genes were selected based on one additional criterion: at least 50% change in mean transcript abundance in at least 1 time point along the time course. The genes were listed based on the order, from top to bottom, in the clustered heat-map shown in Figure 2. Functional sub-/categories, clustering, gene annotations and log_2_(ratio) values are listed. Click here for file

Additional File 4 supplementary_tables_figures. This file contains supplementary tables and figures. Click here for file

Additional File 5 synecho_oligo_sequences. This file contains all of the array oligo probe sequences (sheet 1), including 3064 for *Synechocystis *ORFs, 13 for *E. coli *K-12 ORF controls, and 3 for *Synechocystis *rRNA controls. 104 genes that we failed to design oligo-probes for, and that were excluded from our microarray platform, are listed in sheet 2. Click here for file
