# Supplementary material for: Gene expression patterns of sulfur starvation in Synechocystis sp. PCC 6803
Source: BMC Genomics. 2008 Jul 21;9:344. doi: 10.1186/1471-2164-9-344 (PMC2491639; doi:10.1186/1471-2164-9-344)
Supplement: Additional File 1 — microarray_construction. This supplementary text describes the oligonucleotide probe design and microarray construction and validation. [file 1471-2164-9-344-S1.doc]

## Oligonucleotide probe design and microarray construction

*Supplementary text for:* Zhigang Zhang, Ninad D. Pendse¶, Katherine N. Phillips, James B. Cotner, Arkady Khodursky. 2008. **Gene expression patterns of sulfur deprivation in *Synechocystis* sp. PCC 6803.** *BMC Genomics*, 2008.

## To construct an oligonucleotide-based microarray of the *Synechocystis* protein coding genes, we chose an algorithm that allowed selection of representative long oligonucleotides with high specificity and consistency of hybridization across the set. In the context of microarray design, specificity refers to the inability of the probe to bind strongly to non-target sequences during the hybridization and washing. High specificity can be achieved by avoiding probes with excessive sequence similarity to a non-target sequence that might be present in a complex pool of cellular targets. The hybridization consistency depends on uniformity of probe melting temperatures across the entire collection of the probes, which can be estimated by some computational prediction methods [1].

A freely available software, ArrayOligoSelector [2] developed by Bozdech et al [3], was used to design gene-specific oligonucleotide probes for the entire *Synechocystis* genome. Previously this program had been successfully used to design genome-wide array elements for the human malarial parasitic protozoan *Plasmodium falciparum* [3], the GC rich opportunistic pathogen *Burkholderia cenocepacia* [4] and the butanol biofuel producer *Clostridium acetobutylicum* [5].

Because it was shown that there is a strong correlation between signal intensity and oligo length [6], 70-nt-long oligomers were designed for the *Synechocystis* genome in order to detect genes expressed at low levels. Frequently, one probe per ORF may be sufficient to detect changes in the abundance of a transcript [4, 6, 7]. Thus, one 70-mer per gene was finally designed to reduce the cost. Oligomers for every ORF were selected on the basis of uniqueness within the genome (reduction of cross-hybridization potential), avoidance of significant self-binding (secondary structure), exclusion of low-complexity sequence (minimizing non-specific hybridization), and balanced base composition (consistency in melting temperature). The collection of all possible 70-mer oligonucleotides contained in the coding region of an ORF was used for oligonucleotide selection, which was executed by employing filters for uniqueness, self-binding and complexity, in parallel. The intersections of the set of all oligonucleotides passing these filters were further selected for a desired GC content. We selected oligonucleotides that ranked among the top 5% of unique or almost unique 70-mers in the entire ORF and that were within 5 kcal/mol of a best candidate for the ORF. Next, 33% of top-scoring 70-mers passed the self-binding and low-complexity filters. The GC content cut-off was set to 50%. These filters were applied to the entire set of candidate 70-mers to obtain individual outputs. If no common oligomers were identified, then self binding and complexity filters were relaxed until an intersection appeared. The sequences of oligo-probes are available in a supplementary file [see Additional file 5].

Following computational design and selection of the probes, we constructed the microarray platform in two stages. In the first stage, we produced a 171-probe pilot array and assessed the specificity of spotted probes along with negative control spots by doing single-channel or dual-channel genomic DNA hybridizations. In the second stage, we produced an oligo-array with the genome-wide coverage (representing 3064 out of 3168 protein coding genes) and evaluated its performance based on the ability to detect well-established environmental stress transcriptional responses by two-color hybridization, using the hybridization conditions optimized in the first stage.

## Microarray design stage 1: Pilot array design

In stage one, 158 oligonucleotide probes (about 5% of the genome-wide set) representing well-annotated *Synechocystis* genes were designed. Thirteen *Escherichia coli* K-12 MG1655 oligos were also designed as negative controls. The *E. coli* genes used for designing control probes were selected by doing pair wise BLAST of all the *E. coli* ORFs against all *Synechocystis* genes. With an *E-*value cut-off of 10, 13 *E. coli* ORFs (*b0215, b0273, b0605, b0814, b1078, b1732, b1887, b2798, b2827, b3701, b3744, b4024* and *b4101*), which had a match in *Synechocystis* sequences of at most 19-nt, were selected for designing 70-mer oligos. These 171 oligomers were spotted on glass slides in duplicate for evaluating their specificity using either *Synechocystis* or *E. coli* genomic DNA (gDNA) hybridization. Initially, an *E. coli* cDNA array hybridization protocol was used [8], but there was significant non-specific cross-hybridization as reflected in the ratio of average intensities of target to non-target probes (data not shown). Hence, the protocol was optimized by varying one parameter (salt concentration, hybridization temperature or blocking solution) at a time to minimize the cross-hybridization, along with a slide pre-hybridization step, as described in *Methods*. Finally a significant reduction in cross-hybridization was achieved as the specificity ratio went up from 1.5 to 8-10.

## Table 1 Statistical tests of specificity of hybridization for pilot oligonucleotide arrays of *Synechocystis*.

1. Two class *t*-test.

|  | **Array ID** | **T statistic** | ***P*-value** |
| --- | --- | --- | --- |
| *E. coli* DNA | s-23 | 4.44 | 4.07E-03 |
|  | s-27 | 5.29 | 5.91E-05 |
|  | s-29 | 6.605 | 6.05E-06 |
|  | Average | 6.308 | 1.04E-05 |
| *Synechocystis* DNA | s-24 | -12.79 | 4.23E-29 |
|  | s-26 | -15.1659 | 6.01E-34 |
|  | s-28 | -9.399 | 5.58E-15 |
|  | Average | -15.2287 | 5.06E-31 |

1. Mann-Whitney *U* test.

| **Organism** | **Z statistic** | ***P*-value** |
| --- | --- | --- |
| *E. coli* DNA | -4.12 | <0.0002 |
| *Synechocystis* DNA | -3.76 | <0.0002 |

1. Two class *t*-test on data generated by random sampling.

| **Organism** | **T statistic** | ***P*-value** |
| --- | --- | --- |
| *E. coli* | -23.74 | 1.72E-49 |
| *Synechocystis* | 29.35 | 2.33E-50 |

Subsequent to optimization of the hybridization protocol, we used several statistical tests to assess the performance of the designed oligos in gDNA hybridization experiments. The purpose was to determine whether the intensity of hybridization of target probes was significantly higher than that of non-target probes. First, 6 single-channel genomic DNA hybridizations were performed, 3 with fluorescently labelled *E. coli* DNA and 3 with fluorescently labelled *Synechocystis* DNA. Spots with signal to noise ratios less than two standard deviations away from the background mean were excluded from the analysis. Resulting intensities were subjected to a parametric two-class *t*-test (Table 1A) and non-parametric Mann-Whitney *U* test (Table 1B). We concluded from both tests that the intensity of hybridization of target oligo-probes was significantly higher than that of non-target probes. Because there was an order of magnitude difference in the sample size for target and non-target oligos, 25 random samplings were performed to create two populations of equal sample size from the existing populations. The two-class *t*-test was carried out on this dataset based on the rank of oligo intensity (Table 1C). The average ranks of the two populations were different (both *p*-values were less than 1E-48) and hence, the signals obtained by annealing to the probes from one population had a significantly higher intensity than the other. Figure 1 shows the distribution of mean ranks of intensities from a randomly sampled dataset for *E. coli* (A) and *Synechocystis* (B) DNA hybridization. It is clear that the distribution of average ranks was not overlapping and therefore, the oligos had high selectivity. Finally, two-channel comparative hybridizations (one channel for *E. coli* DNA and the other *Synechocystis* DNA) were done and the log_2_(ratio) for each oligo was calculated. As can be seen in Figure 1C, there was a clear segregation between *E. coli* and *Synechocystis* oligo-probes. Based on these results, the oligo design was deemed to be specific, with no or minimal cross-hybridization.

## Figure 1 Pilot array design to assess probe specificity.

Distribution of mean ranks from randomly generated datasets for single-channel hybridizations with *E. coli* (A) and *Synechocystis* (B) genomic DNA. (C) Two-channel DNA hybridization demonstrates clear separation of the distributions of signals coming from *E. coli* and *Synechocystis* probes based on log_2_(ratio).


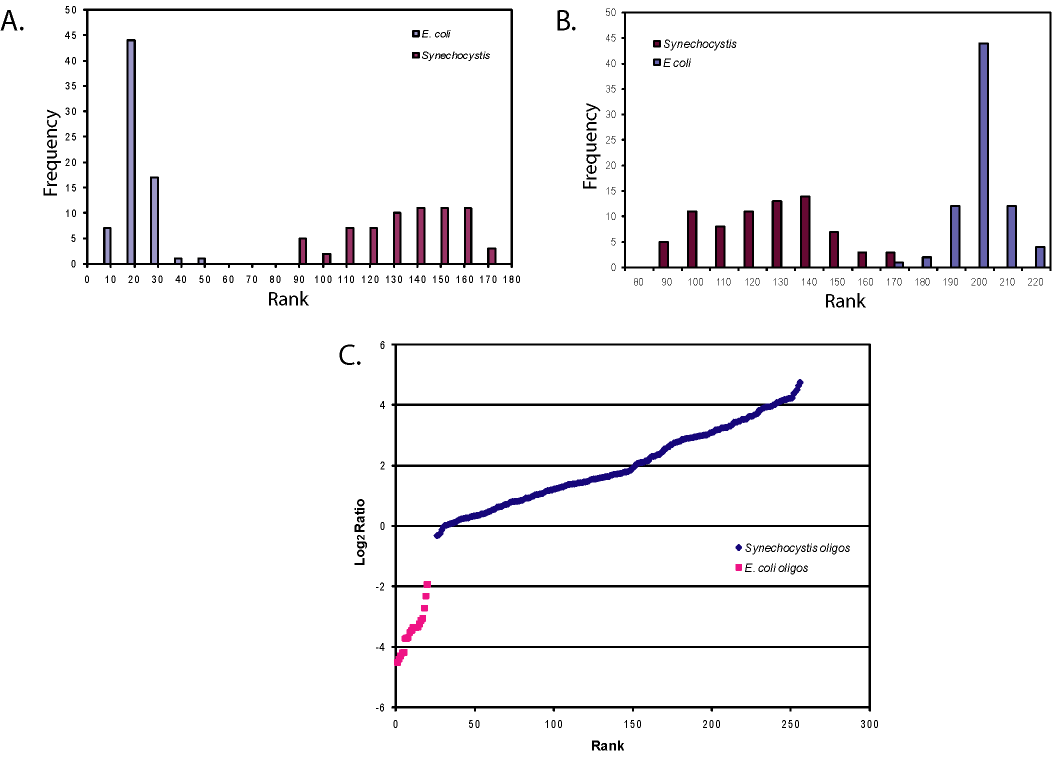


## Microarray design stage 2: Genome-wide array construction

Because sub-genome arrays performed well in our pilot experiments, we concluded that the oligo selection criteria could be used for a large-scale design. We attempted to design 70-mers for all ORFs in the *Synechocystis* genome. Due to significant amount of sequence similarity between some ORFs, we were able to select representative oligonucleotides for 3064 out of 3168 protein coding genes (96.7% coverage). The excluded ORFs (104 total) corresponded to duplicate genes and genes coding for transposases and some other hypothetical proteins [see Additional file 5]. A stringent criterion of no more than 30% identity with the non-target sequence was applied to all oligos. Oligo-probes were also designed for 3 rRNA genes in *Synechocystis*. The layout of the spots was made in such a way as to have 4 control spots, 3 positive and 1 negative, in each of the 16 blocks of the microarray spotted using an in-house robotic printer. A representative genome-wide microarray image is shown in Figure 2.

## Figure 2 Genome-wide *Synechocystis* oligonucleotide (70-mer) DNA microarray. The artificial 16-bit TIFF image of a representative two-color hybridization.


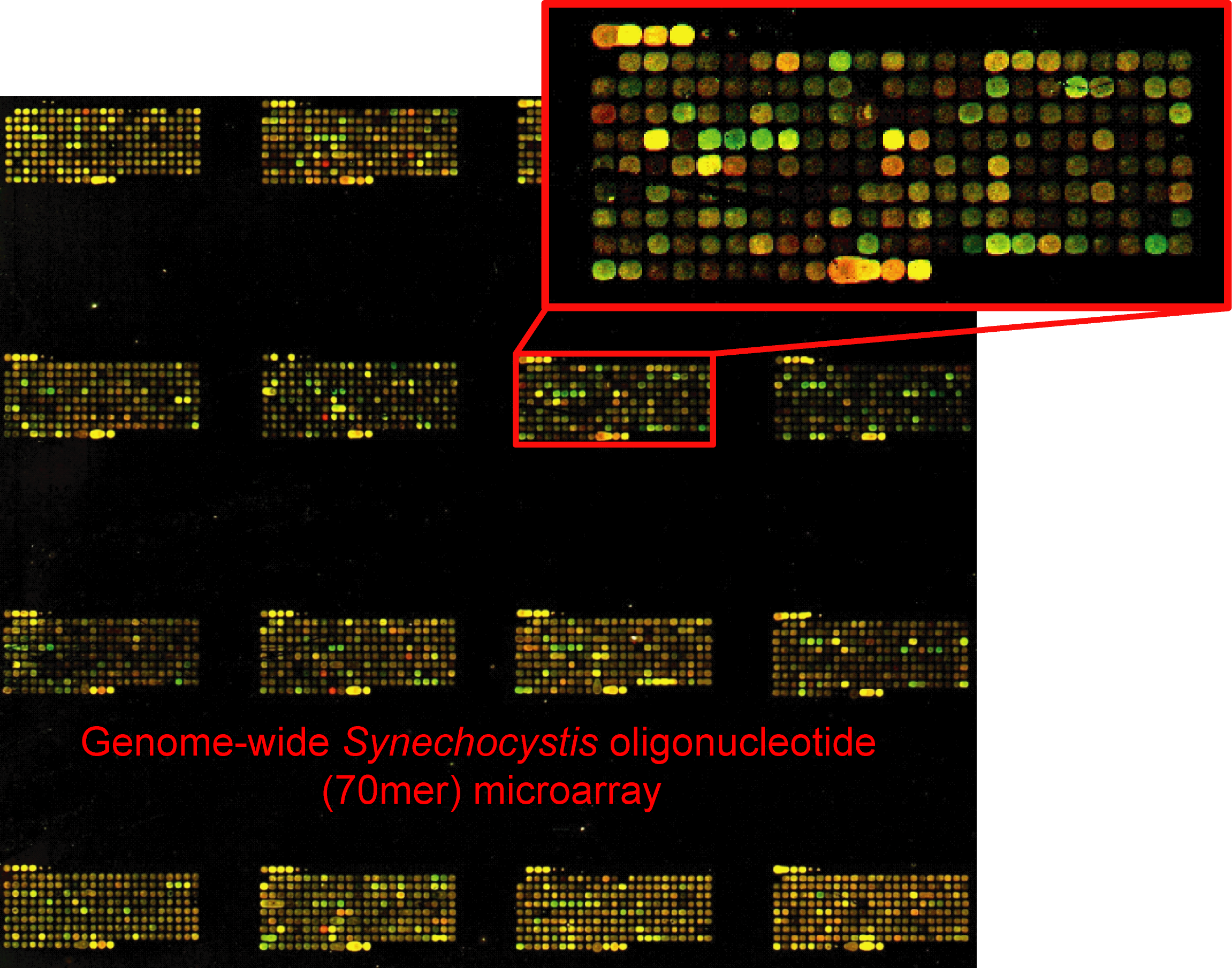


Next, we assessed the performance of the genome-wide array by examining well-known transcriptional responses. First, cells from the mid-exponential growth phase were heat shocked for 1 hour by increasing incubator temperature from 26^o^C to 40 ^o^C. Temperature up-shift usually triggers a heat-shock response [9], which at least in part can be characterized by transcriptional induction of genes encoding for molecular chaperones and proteases. By examining transcriptional responses in 3 independent biological cultures that were shifted to a higher temperature, we identified five known molecular chaperone genes, including *groEL*, *groEL-2*, *groES*, *hspA*, *htpG*, among the top 20 induced genes (mean fold change > 1.5, t-test *P* < 0.05). Induction of those transcripts has been also reported in the studies done by other groups which used cDNA microarrays [10-12]. Second, we tested the array under the condition of salt stress, by subjecting a bacterial culture for 35 min to NaCl at a final concentration of 0.5M. The top 100 induced genes (mean fold change > 1.5, *P* < 0.05) included genes encoding heat shock proteins (*groEL-2*, *hspA*), proteases (*htrA*, *clpB*, *ctpB*, *ftsH*), glucosylglycerol synthetase(*ggpS*), ribosomal proteins (*rps21*, *rpl3*), a sigma factor (*rpoD*), and a high-light-inducible protein (*hliA*). All of them were reported to be inducible by high salt. 11 common genes reported in three different studies were also identified in this study [13-15]. Since the genome-wide original intensity data reported in the literatures are not accessible, more systematic comparisons (overall scatter plot correlation between platforms, overlapping between differentially expressed genes identified by both platforms, etc) cannot be performed.

We also compared the magnitude of change of top ranked common differentially expressed genes identified between this study and literature reports, as shown in Table 2. Literature reports using cDNA microarray platform consistently produced larger values of transcriptional change，from 2 to 30 fold higher than our custom designed long oligonucleotide microarray. The experimental conditions had no significant differences. The main reasons for the magnitude differences could be attributed to differences in array cross-hybridization and data processing. cDNA microarrays use longer and double-strand DNA fragments, which are more prone to non-specific hybridization and can cross-hybridize more easily to similar sequences. This may result in higher background signal than that of oligonucleotide microarrays. On the other hand, those literature reports used local background-subtracted intensities for data normalization. However, there is no theoretical basis for background subtraction. Moreover this procedure may remove some spots with lower spot intensities than background from follow-up analysis, including significantly differentially expressed genes. Based on our own experiences on cDNA microarray data analysis, we prefer to use non-background subtracted intensity data for microarray data preprocessing. Though the magnitude of a particular expression ratio can significantly differ between our oligonucleotide microarray and reported cDNA microarray platform, the relative expression, in terms of rank or “direction” of expression change, appears to be well correlated (Table 2).

**Table 2 Comparison of magnitude of induction folds of common genes between this study and literature reports.** (A) Heat shock. Experimental conditions: this study, 26 to 40°C for 60min; Li et al 2004, 35 to 45°C for 15 min; Suzuki et al 2004&2005, 34 to 44°C for 60min. (B) Salt stress. Experimental conditions: this study, 0.5 M NaCl for 35 min; Kanesaki et al 2002, 0.5 M NaCl for 30 min; Marin et al 2004, 0.684M NaCl for 0.2-24hr; Shoumskaya et al 2005, 0.5 M NaCl for 20 min. Data were presented in mean.

(A)

| Locus(gene) | This study | Li et al 2004 | Suzuki et al 2005&2006 |
| --- | --- | --- | --- |
| slr2075 (*groES*) | 4.4 | 8.7 | 15.5 |
| slr2076 (*groEL*) | 2.7 | 8.2 | 16.4 |
| sll1514 (*hspA*) | 1.7 | 5.1 | 21.6 |
| sll0430(*htpG*) | 1.6 | 9.9 | 3.7 |
| sll0416 (*groEL-2*) | 1.5 | 11.6 | 6.5 |

(B)

| Locus(gene) | This study | Kanesaki et al 2002 | Marin et al 2004 | Shoumskaya et al 2005 |
| --- | --- | --- | --- | --- |
| sll1863 | 13.3 | 52.7 | 265.2 | 106.5 |
| sll1862 | 7.3 | 93.8 | 231.5 | 152.4 |
| sll1514(*hspA*) | 5.1 | 56.2 | 23.9 | 49.7 |
| sll0528 | 3.7 | 40.0 | 50.3 | 74.4 |
| sll1566(*ggpS*) | 3.3 | 10.7 | 7.6 | 13.2 |
| slr1687 | 3.0 | 9.4 | 3.1 | 16.0 |
| ssr2595 | 2.7 | 13.4 | 3.0 | 15.1 |
| slr1544 | 2.4 | 20.3 | 2.1 | 23.2 |
| slr0967 | 2.0 | 16.0 | 25.3 | 32.3 |
| ssl2542(*hliA*) | 1.9 | 5.0 | 2.1 | 9.8 |
| sll1085(*glpD*) | 1.8 | 11.8 | 3.5 | 9.7 |

There are discrepancies among the reports on agreement of inter-lab and inter-platform comparisons of DNA microarray data. Some studies suggest significant disagreement between platforms [16-21], and others show general agreement [22-35]. Systematic analyses indicated that all three platforms (cDNA, long or short oligonucleotide) can give similar and reproducible results if the criterion is the direction of change in gene expression (up-regulation or down-regulation) and minimal emphasis is placed on the magnitude of change [35]. This is also consistent with the notion that microarray experiments are superior in identification of regulatory pattern in genome-wide gene expression rather than giving the researcher quantitative expression values for individual genes [36].

In summary, the extent of concordance between the sets of genes identified in our study and the earlier studies employing PCR-based arrays was consistent with what can be expected from microarray results obtained on different platforms and by different groups. Based on these results, we concluded that the genome-wide array was ready for studying transcriptional responses of the *Synechocystis* genome to poorly understood environmental challenges.

**Methods**

## Probe design and microarray production

## The 70-nt oligomers were designed using ArrayOligoSelector [2] as detailed in the text. The oligos were synthesized by Invitrogen Corp. (Carlsbad, CA, USA), resuspended in 5µl of 3×SSC to a final concentration of 66.7 pmol/µl and spotted onto poly-L-lysine coated microscopic glass slides using OmniGrid Microarray printer (GeneMachines, San Carlos, CA, USA), as described previously [37]. All oligo sequences are provided [see Additional file 5].

## Genomic DNA preparation and fluorescent labelling

Genomic DNA was isolated from stationary phase cultures of *E. coli* K-12 MG1655 and *Synechocystis* sp. PCC 6803, respectively, using standard phenol-chloroform extraction method [38, 39] with minor modifications. Sucrose was supplemented at a final concentration of 0.5M to 1×TE buffer (10mM Tris, 1mM EDTA, pH 8) to efficiently disrupt *Synechocystis* cell envelop. Purified genomic DNA was sheared by sonication, yielding 300-1000bp long DNA fragments for direct labelling using Klenow fragment of DNA polymerase I. The labelling reaction consisted of 2-5µg of genomic DNA, 5 μg of random hexamer (pdN_6_), 1.5 μl of dNTP mix (0.5 mM dATP, 0.5 mM dCTP, 0.5 mM dGTP and 0.2 mM dTTP), 0.2 mM Cy3- or Cy5-dUTP (GE Healthcare, Piscataway, NJ, USA), 1× Klenow buffer and 6-10 units of Klenow. Reactions were incubated at 37°C for 2 hrs.

**Heat and salt treatment experiment**

*Synechocystis* sp. PCC 6803 was grown in BG-11 medium at 26^o^C supplemented with 8mM NaHCO_3_ and buffered with 10mM HEPES-NaOH to a final pH of 7.4. The cells were grown in 0.5L conical flask with continuous shaking exposed to a full spectrum luminescent lamp with a photon flux density of 25µmol photons m^-2^ s^-1^ in 14:10 light dark cycles, continuously supplied with sterile air containing 1% (v/v) CO_2_. For heat shock experiment, 50ml of mid-exponentially growing cells (OD_730nm_ = 0.6) were collected as control and then heat shock response was induced by increasing the incubator temperature to 40^o^C. Cells were harvested after 60 min at the elevated temperature. For salt stress experiment, 5M NaCl was added to the mid-exponential growth phase cell culture to a final NaCl concentration of 0.5M. The cell samples were taken just before (control) and 35min after NaCl addition. All the experiments were done in biological triplicates. Total RNA preparation, cDNA fluorescent labelling and microarray hybridization were the same as presented in the *Method* section of the main text. The microarray data were processed and analyzed as described in the supplementary text above.

**References**
