# Supplementary material for: Gene expression patterns of sulfur starvation in Synechocystis sp. PCC 6803
Source: BMC Genomics. 2008 Jul 21;9:344. doi: 10.1186/1471-2164-9-344 (PMC2491639; doi:10.1186/1471-2164-9-344)

## Supplementary tables and figures

**Supplementary tables**

**Table S1**

**Gene sets significantly affected by sulfate deprivation as determined by SAM gene-set analysis.** The listed sets satisfied the following significance criteria: FDR < 0.01 and p-value < 0.01.

(**A**)

|  | **S-deprivation (<12hr)** |
| --- | --- |
| *Positive* |  |
|  | Folic acid |
|  | Cobalamin, heme, phycobilin and porphyrin |
|  | Riboflavin |
|  | Membranes, lipoproteins, and porins |
|  | Fatty acid, phospholipid and sterol metabolism |
|  | Respiratory terminal oxidases |
|  | Drug and analog sensitivity |
|  | Transport and binding proteins |
|  | Cell envelope |
| *Negative* |  |
|  | Protein and peptide secretion |
|  | Phosphorus compounds |
|  | Pentose phosphate pathway |
|  | Sugars |
|  | Photosynthesis and respiration |
|  | ATP synthase |
|  | Cytochrome b6/f complex |
|  | Photosystem I |
|  | Photosystem II |
|  | Phycobilisome |
|  | Translation |
|  | Ribosomal proteins: synthesis and modification |
|  | Transcription |
|  | Degradation of RNA |
|  |  |
|  | **S-deprivation (12-24hr)** |
| *Positive* |  |
|  | Thioredoxin, glutaredoxin, and glutathione |
| *Negative* |  |
|  | Photosynthesis and respiration |
|  | Photosystem I |
|  | Phycobilisome |
|  |  |
|  | **S-deprivation (>24hr)** |
| *Positive* |  |
|  | Riboflavin |
|  | Degradation of RNA |
|  | Molybdopterin |
| *Negative* |  |
|  | Biotin |
|  | Photosynthesis and respiration |
|  | Photosystem I |
|  | Photosystem II |
|  | Aminoacyl tRNA synthetases and tRNA modification |

(**B**)

|  | **Normal growth control (<24hr)** |
| --- | --- |
| *Positive* |  |
|  | Detoxification |
|  | Chemotaxis |
|  | Cellular processes |
| *Negative* |  |
|  | Glutamate family / Nitrogen assimilation |
|  | Serine family / Sulfur assimilation |
|  | Nucleoproteins |
|  | Pyridoxine |
|  | Carotenoid |
|  | CO_2_ fixation |
|  | Drug and analog sensitivity |
|  |  |
|  | **Normal growth control (>24hr)** |
| *Negative* |  |
|  | Chaperones |
|  | Central intermediary metabolism |
|  | Polysaccharides and glycoproteins |
|  | Pentose phosphate pathway |

**Table S2** Primer design for real-time qRT-PCR.

| **Gene** | **CYORF** | **Primer sequence** | **GC(%)** | **Amplicon (bp)** | **Gene product** |
| --- | --- | --- | --- | --- | --- |
| *psbA2* | slr1311 | For: caccgccgtattcttgatct | 50 | 115 | photosystem II D1 protein |
|  |  | Rev: gatgttgtgctcagcttgga | 50 |  |  |
| *cpcA* | sll1578 | For: cggaggtagtagccgatgtc | 60 | 113 | phycocyanin alpha subunit |
|  |  | Rev: gcccaagccgtttataacaa | 45 |  |  |
| *nblA* | ssl0453 | For: aggcttggaccaaaagttcc | 50 | 107 | phycobilisome degradation protein NblA |
|  |  | Rev: tgaatccttcgatctcacca | 45 |  |  |
| *cysW* | slr1454 | For: ctcctctacggcaaaaatgg | 50 | 116 | sulfate transport system permease protein |
|  |  | Rev: acgaagggcaaggtaacaaa | 45 |  |  |
| *lexA* | sll1626 | For: cctttgagttcgccaatgac | 50 | 119 | LexA repressor |
|  |  | Rev: agtcgattggaacggttacg | 50 |  |  |
| *hoxH* | sll1226 | For: atttgcaccgccagtaactt | 45 | 103 | hydrogenase subunit of the bidirectional hydrogenase |
|  |  | Rev: tgtgggaaatggctggtatt | 45 |  |  |
| *hoxE* | sll1220 | For: tgggctttatgcaggatttc | 45 | 101 | potential NAD-reducing hydrogenase subunit |
|  |  | Rev: gcccatcctagtggagacaa | 55 |  |  |
| *hypA1* | slr1675 | For: gtttgaagtggtgcgacaaa | 45 | 119 | putative hydrogenase expression/formation protein HypA1 |
|  |  | Rev: aaatccaatcctctggctga | 45 |  |  |
| *hypB1* | sll1432 | For: gagacgttgggcatcgttat | 50 | 109 | putative hydrogenase expression/formation protein HypB |
|  |  | Rev: agccggtaaaactgctctga | 50 |  |  |
| *trpA* | slr0966 | For: ggtcaccgataaaccgattg | 50 | 112 | tryptophan synthase alpha chain |
|  |  | Rev: cttaaccatggcgcttccta | 50 |  |  |
| *lysC* | slr0657 | For: caattatggcattcccttgg | 45 | 111 | aspartate kinase |
|  |  | Rev: ttggcaatttcaagccctac | 45 |  |  |
| *rrn 16Sa* | n.a. | For: ccctttcgctaccctagctt | 55 | 103 | 16S ribosomal RNA |
|  |  | Rev: tgtagcggtgaaatgcgtag | 50 |  |  |

**Supplementary figures**

**Figure S1**

**Comparison of transcriptional profiles between S-deprivation conditions with or without HEPES, and a normal growth control**.

All experiments were done in parallel. The statistically significant changes were first determined by a two-class unpaired *t*-test for each condition. Differentially expressed genes (at least three values with >1.5 fold change) were then hierarchically clustered roughly into 5 groups with distinct expression profiles. Representative genes in each cluster are shown.


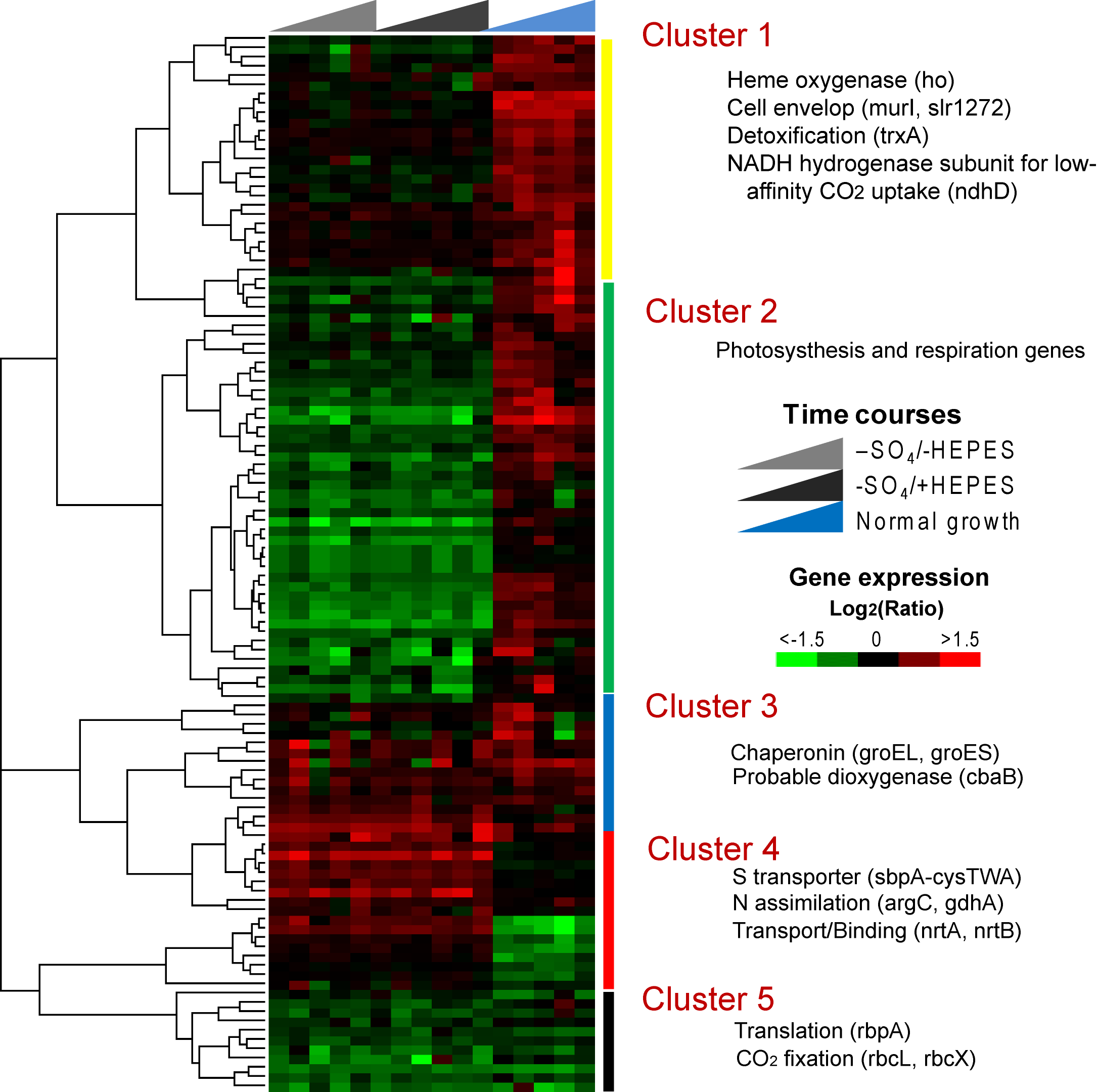


**Figure S2**

**Hydrogen evolution during S-deprivation compared to normal growth control**. Error bars represent s.e.m of triplicate measurements.


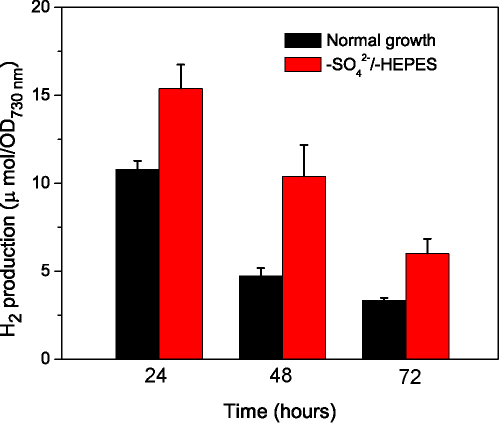

Supplement: Additional File 4 — supplementary_tables_figures. This file contains supplementary tables and figures. [file 1471-2164-9-344-S4.doc]
